# Use of mobile health units in conflict settings—a scoping review

**DOI:** 10.1186/s12913-025-12443-z

**Published:** 2025-03-19

**Authors:** Sarah Awad, Sarika Sheerazi, Johan von Schreeb

**Affiliations:** Department of Global Public Health, Karolinska Institutet, 171 77 Solnavägen, Stockholm, Sweden

**Keywords:** Mobile health units, Armed conflicts, Wars, Disaster medicine

## Abstract

**Background:**

Mobile Health Units (MHUs) provide critical healthcare to underserved populations, however, their effectiveness in conflict settings remains unexplored. This study aims to elucidate the use of MHUs in conflict settings as described in the literature.

**Methods:**

We conducted a scoping review across twenty-three databases, including publications in English between 2000–2022 detailing MHU practices and characteristics in conflict settings. Results were analyzed using thematic content analysis guided by the World Health Organization minimum standards.

**Results:**

Over 7000 documents were screened, yielding 15 publications eligible for inclusion. The included publications comprised 8 peer-reviewed articles, 6 evaluation reports, and 1 master thesis. The predominant study design was mixed methods. Key themes included: operational strategies, key characteristics, services, staff, predeployment preparedness, community engagement, and safety and security. The literature highlights MHUs as flexible resources for bridging health service gaps, noting limitations in sustainability and logistics. Coordination with local health facilities and communities emerged as important for MHU implementation.

**Conclusions:**

Data on MHUs in conflict settings is scarce with inconsistent reporting of key aspects, underscoring the need for improved reporting practices. More studies are needed to understand the role of MHUs in conflict settings.

**Supplementary Information:**

The online version contains supplementary material available at 10.1186/s12913-025-12443-z.

## Introduction

Disasters, whether natural, or manmade including armed conflicts, disrupt societies [1]. They lead to losses that surpass the affected community's ability to manage with its own resources, compelling the need for external assistance [1]. Over the past decade, the number of armed conflicts and populations affected have increased, contributing to 80% of global humanitarian needs [2]. Conflicts are dynamic and unpredictable, marked by recurrent outbreaks of violence of varying intensity [3]. Consequently, boundaries between settings may be unclear, and post-conflict areas could still face violence even after a political settlement has been reached [3]. Challenges in these settings include increased healthcare needs combined with reduced access due to security concerns, infrastructure damage, lack of staff and resource shortages [4, 5]. Studies emphasize that preexisting vulnerability significantly influences civilian mortality with indirect effects exceedingly impacting morbidity, especially non communicable diseases, in addition to direct trauma [6, 7].

International health care assistance may be deployed to aid populations affected by disasters. However, this assistance has been criticized for arriving too late [8], not staying long enough, being uncoordinated [9] and overfocused on trauma care [10]. To improve the shortcomings of international health care response following disasters, the World Health Organization (WHO) initiated the Emergency Medical Teams (EMT) initiative [11, 12]. A set of minimum standards for EMTs, known as the “Blue Book”, and a verification system were developed to enhance the quality, timeliness, and coordination [12]. EMTs, are classified into four types (I-IV), with type I providing outpatient care which can be provided through either fixed or mobile facilities. For EMTs operating in conflicts and insecure environments, additional guidance is provided in the “Red Book”, which emphasizes humanitarian principles and ensuring safety while adhering to International Humanitarian Law (IHL) [13].

Mobile Health Units (MHUs) are broadly defined as intermittent ambulatory health services [14] that provide outpatient care across multiple locations. While comparable to EMT Type 1 mobile, MHUs may encompass a broad range of services extending beyond the scope of EMT Type 1 as defined by WHO [12]. The term 'Mobile Health Units' is often used interchangeably with 'mobile clinics', which typically deliver care inside vehicles, although MHUs can also operate through other forms of transport or temporary setups. Historically, mobile and far-forward facilities have played a critical role in conflict settings and military operations, providing rapid, adaptable care close to the front lines and improving survival rates in austere conditions [15]. MHUs have been described as flexible resources to serve hard-to-reach populations, and emphasis is placed on the ability to refer patients to fixed facilities and higher levels of care [12, 14]. However, they have received criticism regarding irregular service provision, cost-effectiveness concerns, and logistical issues [14].

Amidst the escalation of violent conflicts, MHUs have emerged as critical resources for reaching dispersed populations [14, 16]. Despite their extensive use in disasters, their usefulness in addressing health needs in conflict settings remains inadequately understood [17]. A systematic review conducted by McGowan et al. (2020) on mobile clinics in humanitarian emergencies identified only five studies eligible for inclusion [17]. While the reviewed studies suggest that mobile clinics may enhance service delivery, their limited dataset and general focus on humanitarian emergencies offer few insights into the specific contributions of MHUs in conflict settings. Use of inadequate or inaccurate data may misguide future relief efforts, rendering them ineffective or even harmful to the populations they aim to serve [18]. Addressing these gaps requires a broader exploration of available literature, focusing on key aspects of MHU deployment in conflict-affected areas to provide a more comprehensive understanding of their operational strategies and challenges. To guide the effective utilization of mobile health units in conflict settings, more knowledge is needed.

The aim of this study was to elucidate the use of mobile health units in conflict settings as described in the literature.

## Materials and methods

This scoping review builds on prior work by incorporating a broader range of sources to map existing knowledge and identify gaps in the literature. Given the scarcity of peer-reviewed publications on MHUs in conflict zones, gray literature, such as reports from non-governmental organizations (NGOs) and field evaluations, is included to offer potential insights into operational practices that are often undocumented. This study was conducted employing the framework of Arksey and O’Malley [19], applying the updated methodological guidelines produced by the Joanna Briggs Institute (JBI) Scoping Review Network [20]. A PRISMA checklist for scoping reviews is provided in Additional file 1.

### Search terms

A preliminary search was conducted in September 2021 on PubMed, Web of Science and Google to identify relevant Medical Subject Headings (MESH), keywords, and sources by reviewing studies within the field. The search strategy was developed during the preliminary search in collaboration with a medical librarian at Karolinska Institute and further refined through discussion with the other researcher in the sibling project. The search terms were divided into two categories. Group A included mobile health units and associated MeSH terms and synonyms. Group B included “Armed Conflicts” along with associated MeSH terms, synonyms and the World Bank’s list of Fragile and Conflict Affected Situations (FCAS) [21]. FCAS was included in Group B as the preliminary search revealed that many articles in conflict settings used the name of the country or region rather than explicit conflict-related terms.

### Search strategy

The Campbell Collaboration, CINAHL Cochrane, EMBASE, MEDLINE, PsycINFO, PubMed, and Web of Science databases were queried, combining search terms from the two groups. The search syntax was adapted to the database format while maintaining the search structure, searching “All Fields”, “All Text” or equivalent. All records published between 2000–2022 available in full text in English were exported into EndNote. The final search was conducted 4 January 2023.

The search for gray literature was conducted using the following databases and websites related to disaster response: Evidence Aid, Global Health Observatory, Global Index Medicus, Google Scholar, Health Data Vizhub, Humanitarian Data Exchange, Humanitarian Health Ethics Research Group (HHE), International Committee of the Red Cross (ICRC), International Federation of Red Cross and Red Crescent Societies (IFRC), Médecins Sans Frontières (MSF) Analysis, MSF Centre de Réflexion sur l'Action et les Savoirs Humanitaires (CRASH), MSF Research Unit on Humanitarian Stakes and Practices (UREPH), Open Grey, Prevention Web, Reliefweb, and United Nations Office for the Coordination of Humanitarian Affairs (UNOCHA). For databases with advanced search engines, the same search strategy was applied as for bibliographic databases. For most gray literature sources, Group A (MHU-related) search terms were individually queried in each search engine due to limited advanced search options and inability to combine terms. The search strategy for Google Scholar was adapted to accommodate its limitations in character restrictions and lack of truncation capability. Search terms in Groups A and B were divided into smaller subgroups and combined. The first 300 results were screened for Group A terms paired with conflict-related terms, and the first 100 results for Group A and FCAS pairings. These number were chosen to capture the most relevant hits while ensuring a manageable screening volume. The complete search strategy for all databases as applied to each search engine is provided in Additional file 2. Furthermore, the reference lists of McGowan et al.’s review [17] and the publications included in this review were screened for additional relevant documents. Two experts within the field were consulted for additional sources and reports.

### Eligibility assessment

In alignment with descriptions of mobile health units identified in the primary search [14, 16, 17], MHUs were broadly defined as medical teams delivering outreach health services across multiple locations. Studies that only mentioned MHUs as part of an intervention, or solely reported the number of MHUs or beneficiary demographics were excluded, as such studies lacked sufficient information on the structure, operation, or impact of MHUs. An overview of the eligibility criteria is displayed in Table 1.

**Table 1:** Eligibility criteria

Category	Inclusion	Exclusion
Publication type	Empirical studies (quantitative or qualitative), operational reports, or deployment assessments	Documents with limited traceability (e.g., news articles and blog posts), non-original research (e.g. reviews, commentaries)
Content	Detailing MHU practices, strategies or characteristics	Studies on M-health, insufficient data (e.g. solely reporting on MHU presence or number of beneficiaries)
Language	Full text in English	
Year of publication	2000 – 2022	
Setting	Conflict-affected areas, including active and post-conflict settings	Non-conflict settings, natural disaster responses, rural or nomadic communities
Population	Conflict-affected populations	Refugees and IDPs in stable, non-conflict areas
Operational characteristics	MHU providing outpatient care in more than one location during the deployment	Fixed facilities, inpatient care (hospitals, surgical units) and community-based non-medical care

### Screening process

The initial screening of titles and abstracts was conducted by author SA, employing a liberal inclusion strategy to capture all potentially relevant studies detailing health care provision in conflict settings and FCAS. To ensure consistency, authors SA and SS conducted a pilot dual-screening of records from searches on PubMed and IFRC, achieving an agreement rate of over 90% in inclusion decisions. Full-text screening and assessment were independently performed by authors SA and SS, with discrepancies resolved through discussion, with author JvS serving as a third reviewer to arbitrate conflicts. References were managed using EndNote Online.

### Data extraction and analysis

Data extraction and thematic content analysis were collaboratively undertaken by authors SA and SS. The findings were analyzed using thematic content analysis [22]. Relevant text pertaining to the use of MHUs was entered into an Excel spreadsheet and categorized into different themes. The themes were subsequently compared to WHO guidelines [12, 13] to ascertain standardized terminology and facilitate categorization. If corresponding WHO indicators were not identified, similar aspects of MHU operations were grouped into themes and titled with a suitable name reflecting their content.

## Results

**Fig. 1:**
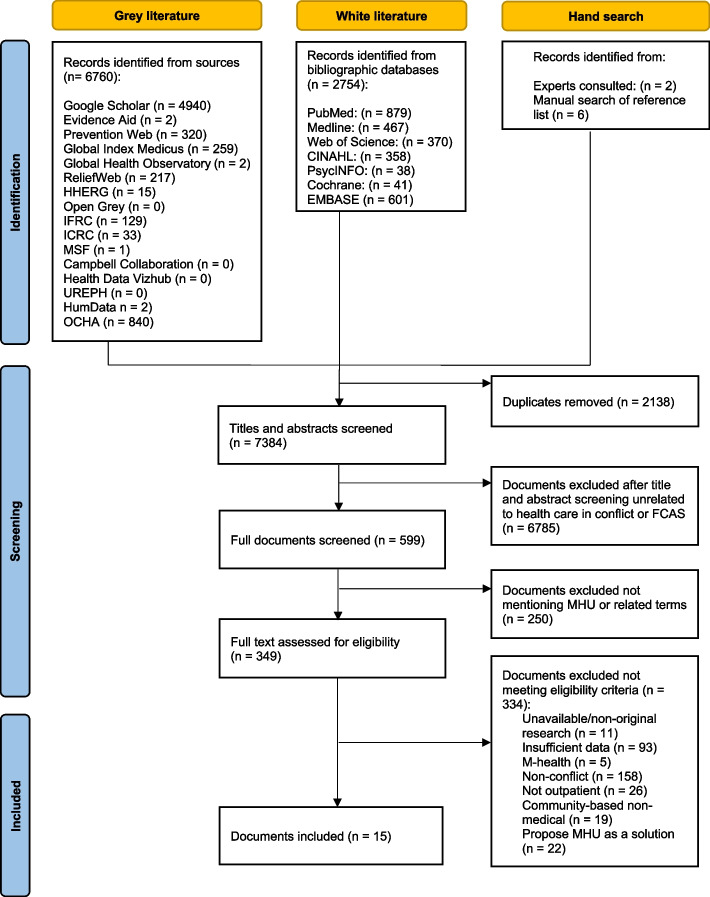
PRISMA Flow Chart

The search yielded 7384 documents for screening, resulting in 15 publications eligible for inclusion. An overview of the screening process is displayed in the PRISMA flow chart in Fig. 1.

### Source characteristics

The included publications reported on mobile health units with the objective of providing health care to populations in conflict-affected areas with limited access to healthcare. The literature reviewed MHUs working in different settings in 9 countries: Afghanistan, Cameroon, Democratic Republic of Congo, Iraq, Palestine, South Sudan, Syria, Uganda and Ukraine. Of the 15 included publications, 8 were peer-reviewed articles, while the remaining included 6 evaluation reports and 1 master thesis. The predominant study design was mixed methods (46.7%), followed by operational overviews (26.7%), longitudinal studies (13.3%), and cross-sectional studies (13.3%). The source characteristics are displayed in Table 2.

**Table 2:** Source characteristics

Lead author	Year	Type of publication	Study design	Focus of care	Geographical location	Target population	Organization type
Abujaber [23]	2021	Peer-reviewed article	Mixed-method case study	Multiple	Syria	Multiple affected	NGO
Afghanistan Red Crescent Society [24]	2006	Evaluation report	Mixed-method program evaluation	Multiple	Afghanistan	Multiple affected	NGO
Al-Halaweh [25]	2019	Peer-reviewed article	Longitudinal study	NCD	Occupied Palestinian territories	Adults with type II diabetes	NGO
Borodin [26]	2020	Evaluation report	Field operations overview	Primary health care	Ukraine	Multiple affected	NGO
Casey [27]	2013	Peer-reviewed article	Cross-sectional study	Family planning	Uganda	Women of reproductive age	NGO
Dulacha [28]	2022	Peer-reviewed article	Mixed-method case study	Multiple	South Sudan	Multiple affected	Governmental
Edmond [29]	2020	Peer-reviewed article	Cross-sectional study	Maternal and child health	Afghanistan	Mothers and children in remote regions	Governmental
Global Response Management [30]	2017	Evaluation report	Field operations overview	Initial trauma care	Iraq	Combatants and civilians near the frontline	NGO
Haneef [31]	2012	Master thesis	Mixed-method program evaluation	Primary health care	Afghanistan	Multiple affected	NGO
Kohli [32]	2012	Peer-reviewed article	Mixed-method case study	SGBV care	Democratic Republic of the Congo	SGBV survivors and their partners	NGO
Morikawa [33]	2011	Peer-reviewed article	Longitudinal study	Primary health care	Afghanistan	Multiple affected	NGO
Omam [34]	2021	Peer-reviewed article	Mixed-method case study	Primary health care and HIV services	Cameroon	Multiple affected	N/A
Qarizada [35]	2019	Evaluation report	Field operations overview	Reproductive, maternal and child health and nutrition	Afghanistan	Pregnant women and children under five years of age	NGO
Spiegel [36]	2018	Evaluation report	Mixed-method case study	Initial trauma care	Iraq	Combatants and civilians near the frontline	NGO
World Health Organization [37]	2019	Evaluation report	Field operations overview	Initial trauma care	Occupied Palestinian territories	Multiple affected in the mass demonstrations	Mixed (governmental and NGO)
Frequencies (n/%)							
2000–2005 (*n* = 0, 0.0%) 2006–2010 (*n* = 1, 6.7%) 2011–2015 (*n* = 4, 26.7%) 2016–2022 (*n* = 10, 66.7%)		Peer-reviewed articles (*n* = 8, 53.3%) Evaluation reports (*n* = 6, 40.0%) Master thesis (*n* = 1, 6.7%)	Mixed-methods studies (*n* = 7, 46.7%) Operational overviews (*n* = 4, 26.7%) Cross-sectional studies (*n* = 2, 13.3%) Longitudinal studies (*n* = 2, 13.3%)	Multiple (*n* = 4, 26.7%) Primary health (*n* = 3, 20%) Trauma (*n* = 3, 20%) Specialized care (*n* = 5, 33.3%)	Afghanistan (*n* = 5, 33.3%), Iraq (*n* = 2, 13.3%), Occupied Palestinian Territories (*n* = 2, 13.3%), remaining locations, including Cameroon, DRC, South Sudan, Syria, Uganda, and Ukraine (each *n* = 1, 6.7%)	Multiple affected (*n* = 10, 66.7%) Women and children (*n* = 3, 20.0%) Targeted subgroups (*n* = 2, 13.3%)	NGO (*n* = 11, 73.3%) Governmental (*n* = 2, 13.3%) Mixed (*n* = 1, 6.7%)

*IDP* Internally displaced people, *MoH* Ministry of Health, *N/A* Not available/applicable, *NCD* Non-communicable disease, *NGO* Non-governmental organization, *SGBV* Sexual and gender-based violence, *DRC* Democratic Republic of Congo.

### Analysis of key findings

The final categorization of the extracted data resulted in seven themes: services, operational strategies, key characteristics, staff, community engagement, safety and security, and predeployment preparedness. The themes are examined in detail in the following sections. Table 3 provides an overview of the reporting frequency of each theme.

**Table 3:** Reporting frequency of themes

Themes	Studies (*n* = 15)	Sources
Services	14 (93,3%)	Abujaber et al. (2021) [23], Al-Halaweh et al. (2019) [25], Borodin et al. (2020) [26], Casey et al. (2013) [27], Dulacha et al. (2022) [28], Edmond et al. (2020) [29], Global Response Management (2017) [30], Haneef (2012) [31], Kohli et al. (2012) [32], Morikawa et al. (2011) [33], Omam et al. (2021) [34], Qarizada et al. (2019) [35], Spiegel et al. (2018) [36], World Health Organization (2019) [37]
Operational strategies	13 (86,7%)	Abujaber et al. (2021) [23], Borodin et al. (2020) [26], Casey et al. (2013) [27], Dulacha et al. (2022) [28], Edmond et al. (2020) [29], Global Response Management (2017) [30], Haneef (2012) [31], Kohli et al. (2012) [32], Morikawa et al. (2011) [33], Omam et al. (2021) [34], Qarizada et al. (2019) [35], Spiegel et al. (2018) [36], World Health Organization (2019) [37]
Key characteristics	11 (73,3%)	Abujaber et al. (2021) [23], Al-Halaweh et al. (2019) [25], Borodin et al. (2020) [26], Casey et al. (2013) [27], Dulacha et al. (2022) [28], Global Response Management (2017) [30], Haneef (2012) [31], Kohli et al. (2012) [32], Omam et al. (2021) [34], Spiegel et al. (2018) [36], World Health Organization (2019) [37]
Staff	14 (93,3%)	Abujaber et al. (2021) [23], Afghanistan Red Crescent Society (2006) [24], Al-Halaweh et al. (2019) [25], Borodin et al. (2020) [26], Casey et al. (2013) [27], Dulacha et al. (2022) [28], Edmond et al. (2020) [29], Global Response Management (2017) [30], Haneef (2012) [31], Kohli et al. (2012) [32], Morikawa et al. (2011) [33], Omam et al. (2021) [34], Qarizada et al. (2019) [35], World Health Organization (2019) [37]
Community engagement	12 (80%)	Abujaber et al. (2021) [23], Al-Halaweh et al. (2019) [25], Borodin et al. (2020) [26], Casey et al. (2013) [27], Dulacha et al. (2022) [28], Global Response Management (2017) [30], Haneef (2012) [31], Kohli et al. (2012) [32], Omam et al. (2021) [34], Qarizada et al. (2019) [35], Spiegel et al. (2018) [36], World Health Organization (2019) [37]
Safety & security	9 (60%)	Abujaber et al. (2021) [23], Borodin et al. (2020) [26], Dulacha et al. (2022) [28], Global Response Management (2017) [30], Haneef (2012) [31], Kohli et al. (2012) [32], Omam et al. (2021) [34], Qarizada et al. (2019) [35], Spiegel et al. (2018) [36]
Predeployment preparedness	7 (46,7%)	Abujaber et al. (2021) [23], Borodin et al. (2020) [26], Dulacha et al. (2022) [28], Global Response Management (2017) [30], Kohli et al. (2012) [32], Qarizada et al. (2019) [35], World Health Organization (2019) [37]

### Services

Three main categories of MHUs were identified based on their foci of care and services provided: 1. MHUs focusing on primary health care (PHC) [23, 24, 26, 28, 31, 33, 34], 2. MHUs providing specialized health services [25, 27, 29, 32, 35] and 3. Trauma Stabilization Points (TSP) [30, 36, 37]. TSPs constitute a targeted MHU that focuses on emergency trauma care, rather than holistic PHC. Particularly useful in high-intensity conflicts, TSPs are specialized and require staff and equipment not normally available at MHUs. Both curative and preventative services were offered in the first two categories. The main activities reported on were consultations, clinical examinations, vaccinations, health promotion activities, and adjustment of treatments. Specialized services provided by MHUs included comprehensive diabetes care [25], nutritional services [35], HIV care [34], maternal and child health [29] and health services tailored to survivors of sexual and gender-based violence (SGBV) [32]. Initial trauma care and stabilization were conducted at the TSPs, focusing on airway and hemorrhage control as well as basic fracture management and wound care with tasks including tourniquet application, airway management, needle compression, and splint placement [30, 36, 37].

### Operational strategies

The reviewed publications elucidated the operational strategies of the teams, including methods of health service delivery, such as site selection and mobility of the MHUs [23, 26–37]. The reviewed literature described MHUs visiting areas with poor access to health facilities due to significant travel distance or security concerns. In choosing locations for MHUs to operate in, Abujaber et al. [23] described the need to ensure accessibility for the target population, taking into consideration security of the area and travel route to the MHU as well as sociodemographic factors, ensuring that vulnerable groups and minorities have access to the facilities. Other MHUs, as in reports from South Sudan [28] and Afghanistan [24], were described as being on standby and deployed as part of an emergency or outbreak response.

MHUs visited multiple sites throughout their deployment, with the teams staying at each location for a duration ranging from 1 to 6 days [23, 28, 29, 31]. Several publications described teams returning to the same site according to a set schedule [27–29, 31, 32, 34, 35], revisiting the sites several times a month [32, 34] or every 1–2 months [27, 29, 31, 35]. As reported by Abujaber [23] and Omam [34], the duration of stay and number of revisits depended on the size or number of communities covered. Teams covering larger or densely populated sites reported longer durations of stay and fewer revisits, whereas MHUs covering smaller camps were able to cover more communities and return more frequently [34]. Two studies detailed the time spent traveling between locations for the MHU [23, 31].

The described strategy of TSPs was to locate close to the point of injury to quicker access patients [36, 37], relocating as per needs and security assessments [30, 36]. In Gaza, ambulances were placed on standby near the TSPs to transport severely injured or ill patients to the nearest hospital [37].

### Key characteristics

The literature outlines several key characteristics, including mode of transportation, operational facilities and equipment [23, 25–28, 30–32, 34, 36, 37]. The main mode of transportation reported was by road. One report described MHUs flown into areas with exceptionally difficult terrains to reach vulnerable and isolated communities [28]. The type of vehicle used varied, including vans, pickup trucks, and regular passenger cars.

The vehicle used for transportation was reported to have been used as an operational facility by some teams [23, 26], whereas others employed vehicles solely for transport rather than for service provision. Six articles reported on teams utilizing preexisting facilities to receive patients, with some teams being embedded within fixed health facilities [27, 32, 34] and others utilizing local community structures such as houses or cultural centers [23, 26, 31]. Some set up facilities of their own using tents [23, 30, 37]. In a case-study on MHUs in Syria, Abujaber et al. [23] conducted interviews with local stakeholders exploring various aspects of MHU deployment and implementation. While most participants favored using physical facilities, citing the benefits of space and privacy for examinations, one participant advocated for a blended approach by traveling on a bus large enough to provide space for examinations. This allowed for adaptability, taking into consideration the destruction of infrastructure and unpredictable conditions [23]. In the study by Kohli et al. [32], providing services in close connection to a fixed primary care facility served the purpose of reducing stigma by ensuring that visits to the mobile clinic providing care for SGBV survivors were “*seen as part of normal health services, not services only for raped women*”.

### Staff

The main medical professions were physicians, nurses, and midwives. Other professions in MHUs included water, sanitation and hygiene (WASH) experts, laboratory specialists, nutritionists and public health officers [23–35, 37]. Non-medical personnel included translators, drivers, coordinators, logisticians, and social workers. Several publications highlighted the importance of recruiting culturally sensitive staff [23, 24, 28, 34] as well as female staff [23] to increase acceptance, especially in culturally conservative societies. While highlighting the need for skilled staff with experience of working in conflict settings [23, 26, 32, 34, 36], different sets of skills were described as useful for responding to different emergencies [28, 32].

### Community engagement

Descriptions of community engagement were frequently detailed in the literature [23, 25–28, 30–32, 34–37]. Several studies highlighted the importance of engaging with local leaders and authorities [23, 26, 28, 31, 32, 35]. Communication and coordination with community leaders supported the teams in need assessments, site selection and route planning, provided operational facilities and accommodations for team members as well as continuous feedback enabling teams to adapt their work as needed. In Ukraine, MHUs conducted visits in coordination with local physicians which reportedly increased the relevance of care by connecting patients to health services rather than temporarily filling gaps in healthcare provision [26].

The use of community health workers (CHWs) was described in six studies as a contributing factor to increasing the utilization of MHUs [23, 27, 28, 32, 35, 37]. CHWs reportedly facilitated raising awareness, gaining acceptance and supporting teams through various tasks. To ensure the long-term viability of interventions, capacity-building initiatives were implemented by some teams, encompassing health education and training of local personnel and CHWs [25, 27, 28, 32, 34, 36, 37]. Although some MHUs provided services aimed at strengthening local health systems, several publications highlight the need for more sustainable interventions [23, 24, 26, 28, 35].

### Safety and security

MHUs reportedly encountered several challenges operating in unstable environments with varying degrees of security threats [23, 26, 28, 30–32, 34–36]. The insecure environment was described to negatively affect the sustainability of staff, opening hours, and accessibility [23, 26, 28, 31, 34], compelling teams to reschedule visits or abstain from working in certain areas [26, 28, 34]. Two studies reported staff being threatened and attacked in some areas, and several service delivery points were reportedly inactive due to security threats [28, 31].

In Mosul, TSPs worked alongside military forces, ensuring security for the teams and enabling access to patients [30, 36]. Some teams opted not to co-locate with Iraqi forces to avoid compromising humanitarian principles. However, these teams faced challenges accessing patients as casualties were brought to the teams by military vehicles, and independently operating teams were stationed further away from the frontline [36]. This operational strategy was controversial, and discussions were raised regarding the responsibility to provide medical care close to the frontline and what implications co-locating with the military might have on future work and safety of MHUs operating in conflict settings [36].

### Predeployment preparedness

Predeployment preparedness encompassed medical training of staff as well as operational, ethical and psychological preparations tailored to the context and target population [23, 26, 28, 30, 32, 37]. While two articles provided detailed descriptions of the training components [26, 28], the remaining only briefly mentioned their occurrence. Three publications noted insufficient preparedness of the teams, particularly concerning relevant conditions, mental health, and contextual aspects, given the challenging and unstable environment in which they were deployed [33, 35, 36]. One report suggested that the training should be carried out by local institutions to ensure relevancy and adherence to local protocols [26].

### Strengths, challenges and recommendations

MHUs reportedly increased access to health services and helped fill critical gaps in areas lacking healthcare facilities or services. Sustained scheduled visits by MHUs were shown to increase uptake of services by increasing acceptance and trust among members of the community served [28, 29, 32]. TSPs effectively addressed gaps in health care provision by locating closer to the point of injury [30, 36, 37], allowing them to promptly triage, treat and refer patients*.* In the report by Spiegel et al. [36] in Mosul, Iraq, 47% of TSP patients were referred, 48% were treated and discharged and 5% died at the TSP site, with similar percentages observed with the TSPs in Gaza [37]. However, the patients were not tracked as they moved through the referral pathway, limiting conclusions on the effectiveness and outcomes of the interventions [36].

While recognized as valuable resources for health service delivery, several challenges were cited. The literature underlines issues related to limited coverage, irregular service provision and inability to address all health needs [24, 26, 28, 32, 34, 35]. MHUs were described to have limited capacity to cover all sites due to variations in size of area and population as well as significant travel distance [24, 28, 34]. These variations consequently impacted the ability of the MHUs to revisit the sites. Furthermore, some MHUs had limited capacity to offer comprehensive care encompassing both curative and preventative services [23, 24]. This was attributed mainly to financial limitations, logistical challenges and the intermittent nature of MHUs.

The literature identified additional issues associated with MHUs including deficiencies in data reporting [30, 36] and logistical issues. In particular, transportation presents several challenges stemming from rugged terrain, poor road conditions and long travel distances [23, 28, 30, 31, 34, 36]. These challenges, compounded by coordination difficulties [28, 36] and unpredictable security situations, posed significant obstacles in delivering essential supplies and personnel. Despite affiliations with fixed facilities and higher-level care, implementation of referrals suffered due to remote locations and limited transportation options, hindering patients’ movement along the care pathway [28, 36]. Moreover, extended travel hours were reported to adversely affect the timing of healthcare delivery [28] and sustainability of staff [23]. Several studies highlighted the need for a predefined exit strategy as communities became reliant on health services, making it difficult for MHUs to withdraw [23, 26, 35].

Two studies described MHUs as impractical for long-term use due to their high cost [28, 35], with quality and standards of the MHUs suffering due to insufficient or untimely funding [23, 30]. Abujaber et al. [23], described tension between local and international stakeholders, emphasizing the need to reallocate funding based on the conditions on the ground and evolving needs. In two reports, questions were raised regarding the efficiency of MHUs and whether they represented the best use of resources [23, 28].

## Discussion

This scoping review identified 15 publications on the use of mobile health units in conflict settings. The literature displayed considerable variations in study design, context and content. While the findings depict MHUs as valuable, versatile resources for improving healthcare access in conflict settings and bridging gaps in healthcare provision, challenges such as logistical difficulties, limited coverage, and deficient funding mechanisms were noted. Several aspects related to the usefulness and implementation of MHUs remain to be addressed.

### Data limitations and need for standardized reporting

Despite being the first study to include operational reports in a review of MHUs in conflict settings, few reports and other gray literature have been identified, echoing McGowan et al.’s observations regarding the paucity of studies documenting MHU usage [17]. Many documents excluded during the final screening mentioned MHU presence but lacked detailed documentation on practices, reinforcing the ongoing discussion on transparency and accountability in relief work [38, 39]. To better understand the usefulness and practices of MHUs, it is imperative for teams to document their experiences and for organizations deploying MHUs to publish their findings.

A significant portion of the literature comprises non-peer-reviewed sources (46.7%), which provide operational insights but may lack methodological rigor, potentially affecting the reliability of findings. Additionally, the literature reveals inconsistencies in reporting of key aspects, despite availability of numerous protocols [12, 40, 41]. While reporting variability may stem from differences in study focus, ensuring consistent metrics across studies on similar aspects could improve comparability and further bridge reporting gaps. These findings underscore the need for routine data collection and standardized reporting mechanisms using quality indicators.

The predominance of mixed-methods studies (46.7%) and operational overviews (26.7%) reflects the complexity of studying MHUs in conflict settings. Studies included in this review often combine qualitative operational assessments with quantitative service data. In contrast, the lower representation of cross-sectional (13.3%) and longitudinal (13.3%) studies highlights the challenges of conducting structured, long-term evaluations in disaster and conflict settings. The instability and security risks of these environments, compounded by the transient nature and logistical challenges inherent to MHUs, further impede systematic data collection and research efforts.

### Operational Variability and the Role of WHO Guidelines

The WHO Emergency Medical Team (EMT) guidelines provide a broad framework for structuring emergency medical response teams, including mobile EMTs [12]. However, only three articles detailed the work of classified EMTs focused primarily on trauma care [30, 36, 37]. Gaps in reporting and variations in operational characteristics complicate assessment of MHUs' alignment with WHO standards, with many publications lacking details on key indicators, such as operational availability, patient encounter rates, and service documentation. The reviewed literature depicted MHUs operating across diverse contexts with similar operational features noted among MHUs offering comparable services. MHUs providing specialized services operated with existing health facilities [27, 32, 35], aligning with WHO’s descriptions of specialized care teams [12]. In contrast, PHC-focused teams reportedly work more independently as autonomous entities [23, 24, 28, 31, 34]. These findings underscore the need for adaptable operational strategies tailored to service type and context, suggesting that expanded guidelines could enhance MHU relevance and effectiveness across diverse settings. Strategic planning of service configurations, guided by the local burden of disease and crisis-related factors, remains essential for optimizing MHU utilization and addressing healthcare demands effectively.

### Implications for Policy and Future Research

The literature highlights the value of MHUs in areas lacking viable alternatives, especially in high-intensity conflicts, enabling quick adaptation and relocation amidst security threats and a volatile environment. The need for mobility in healthcare provision often arises in response to unstable infrastructure, allowing MHUs to reach conflict-affected areas where fixed facilities may be damaged or inaccessible. While facilitating access to underserved populations, the literature highlights limited capacity of MHUs to deliver routine, comprehensive care [23, 24, 34], primarily attributable to logistical, transportation, and supply chain challenges [14, 23, 31]. This poses challenges to their sustainability, echoing previous critique of the service modality [14, 17]. In contrast to the urgent response needed in sudden-onset disasters [12], armed conflicts are marked by recurring cycles of violence [13], with varying health needs and challenges depending on the operational setting [3]. Given the increasing complexity and prolongment of conflicts, implementing more targeted, sustainable interventions at community level may prove more efficient [3, 5, 24]. The predominance of NGO-led MHUs in the reviewed literature further raises concerns about long-term sustainability, particularly in protracted crises. Consequently, a critical assessment of their suitability across different settings is essential. Moreover, efforts to strengthen local healthcare capacity through health education, CHW training, and capacity building initiatives can help mitigate dependence on MHUs and support continuity of care after their exit.

Building on prior reports of increasing attacks on healthcare [42], this review underscores the critical need for robust security measures and strategic site selection to safeguard MHU personnel and ensure continuity of care. Humanitarian actors increasingly adopt strategies that place medical care near the frontline, as with TSPs in Mosul [30, 36]. The TSPs were co-located with military forces, raising ethical concerns regarding adherence to humanitarian principles. While this strategy improved access for combat-injured civilians, its implications on the safety and future recruitment of medical teams remains unclear, underscoring the evolving and complex role of medical care in modern-day conflicts.

Despite criticisms regarding the costliness of MHUs, the literature lacks descriptions regarding their cost or cost-effectiveness. Reports indicate that insufficient and inflexible funding hinders the effectiveness of MHUs [23, 30], underscoring the importance of ongoing dialog and ability to reallocate resources. Further research comparing the cost-effectiveness of MHUs with fixed clinics and alternative service delivery methods is warranted. To address this gap, future organizations should prioritize transparency in operational cost to facilitate comparisons with fixed clinics or alternative service delivery models. Metrics such as patient volumes, referral rates, and health outcomes, when compared to alternative healthcare interventions, could provide valuable insights into the cost-effectiveness of MHUs. Additionally, studies exploring the experiences of MHU teams and organizations, predeployment preparedness, long-term health outcomes and effectiveness of referral pathways could offer practical guidance to optimize MHU operations in conflict settings. A summary of the key findings and implications for MHUs is presented in Table 4.

**Table 4:** Summary of key findings and implications for Mobile Health Units

Category	Findings	Implications
Merits of MHUs	Provides healthcare in underserved areas Adaptable in unstable environments	An adaptable approach to healthcare delivery for underserved and conflict-affected populations
Limitations of MHUs	Logistical constraints Irregular service provision Limited capacity for comprehensive care	Emphasizes the need for improved logistical support and regular scheduling to improve care capacity and reliability
Barriers to Success	Security risks Staffing shortages and supply chain issues Limited and rigid funding mechanisms	Necessitates careful planning, workforce strategies and flexible resource allocation to mitigate risks and address ethical considerations in volatile environments
Facilitators of Success	Strong community partnerships Integration with local health systems Flexible operational strategies	Highlights the importance of local engagement and tailored strategies to ensure continuity and improve MHU effectiveness
Gaps & Recommendations for Research and Deployments	Insufficient and inconsistent data Vaguely defined exit strategies Limited sustainable interventions	Calls for standardized reporting, well-defined exit strategies, and sustainable models for lasting impact

### Methodological Considerations and Limitations

A scoping review design with a broad research question was employed given the limited research available on MHUs. While the results provide valuable insights into the literature on MHUs in conflict settings, the scoping review methodology lacks formal appraisal of sources and bias assessment. The inclusion of reports and field evaluations, largely from deploying organizations, may introduce reporting bias. To mitigate this, independent monitoring and evaluation of deployments should be considered.

Conducted as part of a sibling project on MHUs in disasters, our focus on natural disasters and conflict settings may have excluded relevant data in studies from other settings applicable to disasters. Moreover, limiting inclusion to English-language publications dated after 2000 might have omitted relevant articles in other languages or older studies. Initial title and abstract screening were conducted by a single reviewer, which may have introduced selection bias. To address this, we applied a liberal inclusion strategy and conducted a pilot dual screening on a subset of articles to ensure consistency and alignment in decision-making.

## Conclusions

Despite their frequent deployment in disasters, there are few published studies and reports detailing the use of mobile health units (MHUs) in conflict settings. Sparse and inconsistent data on MHU utilization, coupled with discrepancies in adherence to WHO guidelines, highlight the need for improved data collection and reporting practices. The reviewed articles describe MHUs as flexible resources for bridging health service gaps but underscore several limitations, particularly related to sustainability and logistics. Tailoring strategies to address local disease burden and contextual challenges can enhance MHU relevance. Coordination and engagement with local health facilities and community members were emphasized as key facilitators for effective implementation. To improve MHU utilization in conflict settings more data are needed. We encourage deployed teams and organizations to publish their findings and evaluations.

## Supplementary Information

Supplementary Material 1.

Supplementary Material 2.

## Data Availability

No datasets were generated or analysed during the current study.

## Abbreviations

CHW: Community Health Worker
CRASH: MSF Centre de Réflexion sur l’Action et les Savoirs Humanitaires
DRC: Democratic Republic of Congo
EMT: Emergency Medical Team
FCAS: Fragile and Conflict-Affected Situations
HIV: Human Immunodeficiency Virus
HHE: Humanitarian Health Ethics Research Group
ICRC: International Committee of the Red Cross
IDP: Internally Displaced Persons
IFRC: International Federation of Red Cross and Red Crescent Societies (IFRC)
MESH: Medical Subject Headings
MHU: Mobile Health Unit
MSF: Médecins Sans Frontières (MSF) Analysis
NCD: Non-communicable Disease
NGO: Non-governmental organization
PHC: Primary Health Care
SGBV: Sexual and Gender-Based Violence
TSP: Trauma Stabilization Point
UNOCHA: United Nations Office for the Coordination of Humanitarian Affairs
UREPH: MSF Research Unit on Humanitarian Stakes and Practices
WASH: Water, Sanitation and Hygiene
WHO: World Health Organization
