# Supplementary material for: Use of mobile health units in conflict settings—a scoping review
Source: BMC Health Serv Res. 2025 Mar 19;25:409. doi: 10.1186/s12913-025-12443-z (PMC11924679; doi:10.1186/s12913-025-12443-z)
Supplement: Supplementary file 2 — Supplementary Material 2. [file 12913_2025_12443_MOESM2_ESM.pdf]

Additional file 2 - Search Queries for Databases

Description:
Complete search queries used for each database and source, outlining keywords and filters applied.

Search terms

<table>
<tr><th>Group A</th><th colspan="2">Group B</th></tr>
<tr><td rowspan="2">"Mobile Health Unit*" OR "Mobile Healthcare Unit*" OR "Mobile Clinic*" OR "Mobile Health Van*" OR "Field Hospital*" OR "Mobile Hospital*" OR "Mobile Medical team*" OR "Foreign medical team*" OR "Emergency medical team*" OR "EMT type 1" OR "Mobile Care Team*" OR "Mobile Health Clinic*" OR "Mobile Health Team*" OR "Outreach Health Service*" OR "Medical Relief"</td><th>Armed Conflict</th><th>FCAS</th></tr>
<tr><td>"Armed Conflict*" OR "War*" OR "Warzone*" OR "War Zone*" OR "Conflict Zone*" OR "Conflict Affected" OR "Conflict Setting*" OR "Man-made Disaster*" OR "Fragile and Conflict-affected Situations" OR "Complex Emergenc*"</td><td>Afghanistan OR Armenia OR Azerbaijan OR "Burkina Faso" OR Burundi OR Cameroon OR "Central African Republic" OR Chad OR Comoros OR "Democratic Republic of Congo" OR "Congo" OR Eritrea OR Ethiopia OR "Guinea-Bissau" OR Haiti OR Iraq OR Kiribati OR Kosovo OR Lebanon OR Libya OR Mali OR "Marshall Islands" OR "Micronesia" OR Mozambique OR Myanmar OR Niger OR Nigeria OR "Papua New Guinea" OR "Solomon Islands" OR Somalia OR "South Sudan" OR Sudan OR "Syria" OR "Timor-Leste" OR Tuvalu OR Venezuela OR "West Bank" OR Gaza OR Yemen OR Zimbabwe</td></tr>
</table>

PubMed
Group A & B combined with AND
Filters applied:

- English Language
- Years 2000-2022

(("Mobile Health Unit*" OR "mobile healthcare unit*" OR "Mobile Clinic*" OR "Mobile Health Van*" OR "Field Hospital*" OR "Mobile Hospital*" OR "Mobile Medical team*" OR "Foreign medical team*" OR "Emergency medical team*" OR "EMT type 1" OR "Mobile Care Team*" OR "mobile health clinic*" OR "mobile health team*" OR "outreach health service*" OR "medical relief") AND ("Armed Conflict*" OR "war" OR "wars" OR "warzone*" OR "war zone*" OR "conflict zone*" OR "conflict affected" OR "conflict setting*" OR "man-made disaster*" OR "fragile and conflict-affected situations" OR "complex emergenc*" OR Afghanistan OR Armenia OR Azerbaijan OR "Burkina Faso" OR Burundi OR Cameroon OR "Central African Republic" OR Chad OR Comoros OR "Democratic Republic of Congo" OR "DRC" OR "Congo-Kinshasa" OR "DR Congo" OR "Congo" OR Eritrea OR Ethiopia OR "Guinea-Bissau" OR Haiti OR Iraq OR Kiribati OR Kosovo OR Lebanon OR Libya OR Mali OR "Marshall Islands" OR "Micronesia" OR Mozambique OR Myanmar OR Burma OR Niger OR Nigeria OR "Papua New Guinea" OR "Solomon Islands" OR Somalia OR "South Sudan" OR

Sudan OR "Syria*" OR "Timor-Leste" OR "East Timor" OR Tuvalu OR Venezuela OR "West Bank" OR Gaza OR Yemen OR Zimbabwe))

PsycInfo
Group A & B combined with AND
Filters applied: English Language and years 2000-2022

("Mobile Health Unit*" OR "mobile healthcare unit*" OR "Mobile Clinic*" OR "Mobile Health Van*" OR "Field Hospital*" OR "Mobile Hospital*" OR "Mobile Medical team*" OR "Foreign medical team*" OR "Emergency medical team*" OR "EMT type 1" OR "Mobile Care Team*" OR "mobile health clinic*" OR "mobile health team*" OR "outreach health service*" OR "medical relief") AND ("Armed Conflict*" OR "war" OR "wars" OR "warzone*" OR "war zone*" OR "conflict zone*" OR "conflict affected" OR "conflict setting*" OR "man-made disaster*" OR "fragile and conflict-affected situations" OR "complex emergenc*" OR Afghanistan OR Armenia OR Azerbaijan OR "Burkina Faso" OR Burundi OR Cameroon OR "Central African Republic" OR Chad OR Comoros OR "Democratic Republic of Congo" OR "DRC" OR "Congo-Kinshasa" OR "DR Congo" OR "Congo" OR Eritrea OR Ethiopia OR "Guinea-Bissau" OR Haiti OR Iraq OR Kiribati OR Kosovo OR Lebanon OR Libya OR Mali OR "Marshall Islands" OR "Micronesia" OR Mozambique OR Myanmar OR Burma OR Niger OR Nigeria OR "Papua New Guinea" OR "Solomon Islands" OR Somalia OR "South Sudan" OR Sudan OR "Syria*" OR "Timor-Leste" OR "East Timor" OR Tuvalu OR Venezuela OR "West Bank" OR Gaza OR Yemen OR Zimbabwe)

Web of Science
Group A & B combined with AND
Filters applied: All Fields, English Language and years 2000-2022

("Mobile Health Unit*" OR "mobile healthcare unit*" OR "Mobile Clinic*" OR "Mobile Health Van*" OR "Field Hospital*" OR "Mobile Hospital*" OR "Mobile Medical team*" OR "Foreign medical team*" OR "Emergency medical team*" OR "EMT type 1" OR "Mobile Care Team*" OR "mobile health clinic*" OR "mobile health team*" OR "outreach health service*" OR "medical relief")

("Armed Conflict*" OR "war" OR "wars" OR "warzone*" OR "war zone*" OR "conflict zone*" OR "conflict affected" OR "conflict setting*" OR "man-made disaster*" OR "fragile and conflict-affected situations" OR "complex emergenc*" OR Afghanistan OR Armenia OR Azerbaijan OR "Burkina Faso" OR Burundi OR Cameroon OR "Central African Republic" OR Chad OR Comoros)) OR ALL=("Democratic Republic of Congo" OR "DRC" OR "Congo-Kinshasa" OR "DR Congo" OR "Congo" OR Eritrea OR Ethiopia OR "Guinea-Bissau" OR Haiti OR Iraq OR Kiribati OR Kosovo OR Lebanon OR Libya OR Mali OR "Marshall Islands" OR "Micronesia" OR Mozambique OR Myanmar OR Burma OR Niger OR Nigeria OR "Papua New Guinea" OR "Solomon Islands" OR Somalia OR "South Sudan" OR Sudan OR "Syria*" OR "Timor-Leste" OR "East Timor" OR Tuvalu OR Venezuela OR "West Bank" OR Gaza OR Yemen OR Zimbabwe)

CINAHL
Group A & B combined with AND
Filters applied: All Text, English Language and years 2000-2022

(("Mobile Health Unit*" OR "mobile healthcare unit*" OR "Mobile Clinic*" OR "Mobile Health Van*" OR "Field Hospital*" OR "Mobile Hospital*" OR "Mobile Medical team*" OR "Foreign medical team*" OR "Emergency medical team*" OR "EMT type 1" OR "Mobile Care Team*" OR "mobile health clinic*" OR "mobile health team*" OR "outreach health service*" OR "medical relief") AND ("Armed Conflict*" OR "war" OR "wars" OR "warzone*" OR "war zone*" OR "conflict zone*" OR "conflict affected" OR "conflict setting*" OR "man-made disaster*" OR "fragile and conflict-affected situations" OR "complex emergenc*" OR Afghanistan OR Armenia OR Azerbaijan OR "Burkina Faso" OR Burundi OR Cameroon OR "Central African Republic" OR Chad OR Comoros OR "Democratic Republic of Congo" OR "DRC" OR "Congo-Kinshasa" OR "DR Congo" OR "Congo" OR Eritrea OR Ethiopia OR "Guinea-Bissau" OR Haiti OR Iraq OR Kiribati OR Kosovo OR Lebanon OR Libya OR Mali OR "Marshall Islands" OR "Micronesia" OR Mozambique OR Myanmar OR Burma OR Niger OR Nigeria OR "Papua New Guinea" OR "Solomon Islands" OR Somalia OR "South Sudan" OR Sudan OR "Syria*" OR "Timor-Leste" OR "East Timor" OR Tuvalu OR Venezuela OR "West Bank" OR Gaza OR Yemen OR Zimbabwe))

Cochrane (21 review, 20 trials)
Group A & B combined with AND
Filters applied: All Text, English Language and Years 2000-2022

(("Mobile Health Unit*" OR "mobile healthcare unit*" OR "Mobile Clinic*" OR "Mobile Health Van*" OR "Field Hospital*" OR "Mobile Hospital*" OR "Mobile Medical team*" OR "Foreign medical team*" OR "Emergency medical team*" OR "EMT type 1" OR "Mobile Care Team*" OR "mobile medical care" OR "mobile health clinic*" OR "mobile health team*" OR "outreach health service*" OR "medical relief" in All Text) AND ("Armed Conflict*" OR "war" OR "wars" OR "warzone*" OR "war zone*" OR "conflict zone*" OR "conflict affected" OR "conflict setting*" OR "man-made disaster*" OR "humanitarian" OR "fragile and conflict-affected situations" OR "complex emergenc*" OR Afghanistan OR Armenia OR Azerbaijan OR "Burkina Faso" OR Burundi OR Cameroon OR "Central African Republic" OR Chad OR Comoros OR "Democratic Republic of Congo" OR "DRC" OR "Congo-Kinshasa" OR "DR Congo" OR "Congo" OR Eritrea OR Ethiopia OR "Guinea-Bissau" OR Haiti OR Iraq OR Kiribati OR Kosovo OR Lebanon OR Libya OR Mali OR "Marshall Islands" OR "Micronesia" OR Mozambique OR Myanmar OR Burma OR Niger OR Nigeria OR "Papua New Guinea" OR "Solomon Islands" OR Somalia OR "South Sudan" OR Sudan OR "Syria*" OR "Timor-Leste" OR "East Timor" OR Tuvalu OR Venezuela OR "West Bank" OR Gaza OR Yemen OR Zimbabwe))

Medline
Group A & B combined with AND
Filters applied:

- English Language
- Years 2000-2022

(("Mobile Health Unit*" OR "mobile healthcare unit*" OR "Mobile Clinic*" OR "Mobile Health Van*" OR "Field Hospital*" OR "Mobile Hospital*" OR "Mobile Medical team*" OR "Foreign medical team*" OR "Emergency medical team*" OR "EMT type 1" OR "Mobile Care Team*" OR "mobile health clinic*" OR "mobile health team*" OR "outreach health service*" OR

"medical relief") AND ("Armed Conflict*" OR "war" OR "wars" OR "warzone*" OR "war zone*" OR "conflict zone*" OR "conflict affected" OR "conflict setting*" OR "man-made disaster*" OR "fragile and conflict-affected situations" OR "complex emergenc*" OR Afghanistan OR Armenia OR Azerbaijan OR "Burkina Faso" OR Burundi OR Cameroon OR "Central African Republic" OR Chad OR Comoros OR "Democratic Republic of Congo" OR "DRC" OR "Congo-Kinshasa" OR "DR Congo" OR "Congo" OR Eritrea OR Ethiopia OR "Guinea-Bissau" OR Haiti OR Iraq OR Kiribati OR Kosovo OR Lebanon OR Libya OR Mali OR "Marshall Islands" OR "Micronesia" OR Mozambique OR Myanmar OR Burma OR Niger OR Nigeria OR "Papua New Guinea" OR "Solomon Islands" OR Somalia OR "South Sudan" OR Sudan OR "Syria*" OR "Timor-Leste" OR "East Timor" OR Tuvalu OR Venezuela OR "West Bank" OR Gaza OR Yemen OR Zimbabwe))

Prevention Web
This search engine, similar to several others, was limited in its capacity to combine multiple search terms or use truncation. Consequently, each term from Group A (terms related to MHUs) was individually queried in both singular and plural forms (e.g., “mobile health unit,” “mobile health units”). For each term, the titles and abstracts/report descriptions of the results were screened.

Evidence Aid
Each search term in group A (MHU-related terms) was individually queried in singular and plural (ex; “mobile health unit”, “mobile health units”, etc) using the default search engine.

Global Index Medicus
Group A & B combined with AND
Filters applied:

- English Language
- Years 2000-2022
- “Title, Abstract, Subject”

((("mobile health unit") OR ("mobile health units") OR ("mobile healthcare unit") OR ("mobile healthcare units") OR ("mobile clinic") OR ("mobile clinics") OR ("mobile health van") OR ("mobile health vans") OR ("field hospital") OR ("field hospitals") OR ("mobile hospital") OR ("mobile hospitals") OR ("mobile medical team") OR ("mobile medical teams") OR ("foreign medical team") OR ("foreign medical teams") OR ("emergency medical team") OR ("emergency medical teams") OR ("EMT type 1") OR ("mobile care team") OR ("mobile care teams") OR ("mobile health clinic") OR ("mobile health clinics") OR ("mobile health team") OR ("mobile health teams") OR ("outreach health service") OR ("outreach health services") OR ("medical relief")) AND (("armed conflict") OR ("armed conflicts") OR ("war") OR ("wars") OR ("warzone") OR ("warzones") OR ("war zone") OR ("war zones") OR ("conflict zone") OR ("conflict zones") OR ("conflict affected") OR ("conflict setting") OR ("conflict settings") OR ("man-made disaster") OR ("man-made disasters") OR ("fragile and conflict-affected situations") OR ("complex emergency") OR ("complex emergencies") OR ("Afghanistan") OR ("Armenia") OR ("Azerbaijan") OR ("Burkina Faso") OR ("Burundi") OR ("Cameroon") OR ("Central African Republic") OR ("Chad") OR ("Comoros") OR ("Democratic Republic of Congo") OR ("DRC") OR ("Congo-Kinshasa") OR ("DR Congo") OR ("Congo") OR ("Eritrea") OR ("Ethiopia") OR ("Guinea-Bissau") OR ("Haiti") OR ("Iraq") OR ("Kiribati") OR ("Kosovo")

OR ("Lebanon") OR ("Libya") OR ("Mali") OR ("Marshall Islands") OR ("Micronesia") OR ("Mozambique") OR ("Myanmar") OR ("Burma") OR ("Niger") OR ("Nigeria") OR ("Papua New Guinea") OR ("Solomon Islands") OR ("Somalia") OR ("South Sudan") OR ("Sudan") OR ("Syria") OR ("Syrias") OR ("Timor-Leste") OR ("East Timor") OR ("Tuvalu") OR ("Venezuela") OR ("West Bank") OR ("Gaza") OR ("Yemen") OR ("Zimbabwe")))

Global Health Observatory
Each search term in group A (MHU-related terms) was individually queried in singular and plural (ex; “mobile health unit”, “mobile health units”, etc) using the default search engine.

HHERG
Each search term in group A (MHU-related terms) was individually queried in singular and plural (ex; “mobile health unit”, “mobile health units”, etc) using the default search engine.

Open grey
Each search term in group A (MHU-related terms) was individually queried in singular and plural (ex; “mobile health unit”, “mobile health units”, etc) using the default search engine.

Campbell
Each search term in group A (MHU-related terms) was individually queried in singular and plural (ex; “mobile health unit”, “mobile health units”, etc) using the default search engine.

Health Data Vizhub
Each search term in group A (MHU-related terms) was individually queried in singular and plural (ex; “mobile health unit”, “mobile health units”, etc) using the default search engine.

UNOCHA
The search engine was limited in its ability to combine different search terms and usage of truncation. Therefore, each search term in group A (MHU-related terms) was individually queried in singular and plural (ex; “mobile health unit”, “mobile health units”, etc). Another limitation of this search engine was the ability to only view the first 10 pages with 10 results on each page despite stating over 1000 results on some terms. Hence, only the first visible 10 pages were screened with maximum of 100 results screened on each search term.

EMBASE (Ovid)
Group A & B combined with AND
Filters applied:

- English Language
- Years 2000-2022

(("Mobile Health Unit*" OR "mobile healthcare unit*" OR "Mobile Clinic*" OR "Mobile Health Van*" OR "Field Hospital*" OR "Mobile Hospital*" OR "Mobile Medical team*" OR "Foreign medical team*" OR "Emergency medical team*" OR "EMT type 1" OR "Mobile Care Team*" OR "mobile health clinic*" OR "mobile health team*" OR "outreach health service*" OR "medical relief") AND ("Armed Conflict*" OR "war" OR "wars" OR "warzone*" OR "war zone*" OR "conflict zone*" OR "conflict affected" OR "conflict setting*" OR "man-made disaster*" OR "fragile and conflict-affected situations" OR "complex emergenc*" OR Afghanistan OR

Armenia OR Azerbaijan OR "Burkina Faso" OR Burundi OR Cameroon OR "Central African Republic" OR Chad OR Comoros OR "Democratic Republic of Congo" OR "DRC" OR "Congo-Kinshasa" OR "DR Congo" OR "Congo" OR Eritrea OR Ethiopia OR "Guinea-Bissau" OR Haiti OR Iraq OR Kiribati OR Kosovo OR Lebanon OR Libya OR Mali OR "Marshall Islands" OR "Micronesia" OR Mozambique OR Myanmar OR Burma OR Niger OR Nigeria OR "Papua New Guinea" OR "Solomon Islands" OR Somalia OR "South Sudan" OR Sudan OR "Syria*" OR "Timor-Leste" OR "East Timor" OR Tuvalu OR Venezuela OR "West Bank" OR Gaza OR Yemen OR Zimbabwe))

IFRC

This search engine allowed for year restrictions. A preliminary search displayed the same results for search terms queried in singular or plural. Each search term in group A (MHU-related terms) was individually queried in singular (ex; "mobile health unit", "mobile healthcare unit", "mobile clinic" etc) using the default search engine.

Filters applied:

- 2000-01-01 -- 2022-12-31

ICRC

A preliminary search displayed the same results for search terms queried in singular or plural. Each search term in group A (MHU-related terms) was individually queried in singular (ex; "mobile health unit", "mobile healthcare unit", "mobile clinic" etc) using the default search engine.

Filters applied:

- Years 2000-2022
- Type of document:
    - Publications
    - Reports

MSF

A preliminary search displayed the same results for search terms queried in singular or plural. Each search term in group A (MHU-related terms) was individually queried in singular (ex; "mobile health unit", "mobile healthcare unit", "mobile clinic" etc) using the default search engine.

Filters applied:

- Format: Report

UREPH

Each search term in group A (MHU-related terms) was individually queried in singular and plural (ex; "mobile health unit", "mobile health units", etc) using the default search engine.

Relief Web

Group A & B combined with AND

Filters applied:

- Language: English
- Years: 2000/01/01 - 2022/12/31
- Content format:
    - Analysis

- Evaluation and Lessons Learned
- Assessment

(("mobile health unit" OR "mobile health units" OR "mobile healthcare unit" OR "mobile healthcare units" OR "mobile clinic" OR "mobile clinics" OR "mobile health van" OR "mobile health vans" OR "field hospital" OR "field hospitals" OR "mobile hospital" OR "mobile hospitals" OR "mobile medical team" OR "mobile medical teams" OR "foreign medical team" OR "foreign medical teams" OR "emergency medical team" OR "emergency medical teams" OR "EMT type 1" OR "mobile care team" OR "mobile care teams" OR "mobile health clinic" OR "mobile health clinics" OR "mobile health team" OR "mobile health teams" OR "outreach health service" OR "outreach health services" OR "medical relief") AND ("armed conflict" OR "armed conflicts" OR "war" OR "wars" OR "warzone" OR "warzones" OR "war zone" OR "war zones" OR "conflict zone" OR "conflict zones" OR "conflict affected" OR "conflict setting" OR "conflict settings" OR "man-made disaster" OR "man-made disasters" OR "fragile and conflict-affected situations" OR "complex emergency" OR "complex emergencies" OR Afghanistan OR Armenia OR Azerbaijan OR "Burkina Faso" OR Burundi OR Cameroon OR "Central African Republic" OR Chad OR Comoros OR "Democratic Republic of Congo" OR "DRC" OR "Congo-Kinshasa" OR "DR Congo" OR "Congo" OR Eritrea OR Ethiopia OR "Guinea-Bissau" OR Haiti OR Iraq OR Kiribati OR Kosovo OR Lebanon OR Libya OR Mali OR "Marshall Islands" OR "Micronesia" OR Mozambique OR Myanmar OR Burma OR Niger OR Nigeria OR "Papua New Guinea" OR "Solomon Islands" OR Somalia OR "South Sudan" OR Sudan OR "Syria*" OR "Timor-Leste" OR "East Timor" OR Tuvalu OR Venezuela OR "West Bank" OR Gaza OR Yemen OR Zimbabwe))

Humdata
Each search term in group A (MHU-related terms) was individually queried in singular and plural (ex; “mobile health unit”, “mobile health units”, etc) using the default search engine.

Google Scholar
Regarding Google Scholar, we adapted the search strategy to account for its specific limitations, such as the 256-character restriction and the lack of truncation capability. To manage this, the initial two search groups were divided into smaller subgroups.

By default, Google Scholar searches all terms in full text. A preliminary search was conducted to identify effective terms by pairing each search term in Group A (MHU-related terms) with armed conflicts and related terms from Group B. For this initial assessment, the first 50 results were screened, focusing on terms that consistently generated relevant results. The finalized MHU search terms included “Mobile Health Unit”, “Mobile Health Units”, “Mobile Clinic”, “Mobile Clinics”, “Mobile Medical Team”, “Mobile Medical Teams”, "Mobile Health Clinic", "Mobile Health Clinics", "Mobile Health Team", "Mobile Health Teams", “Foreign Medical Team”, “Foreign Medical Teams”, “Emergency Medical Team”, “Emergency Medical Teams” and “EMT type 1”.

The refined search terms for Mobile Health Units (MHUs) were then paired separately with terms for armed conflicts and for Fragile and Conflict-Affected Settings (FCAS). Given the character limit, the finalized MHU terms were organized into three groups and paired separately with terms for armed conflicts which were separated into two groups.

The groups:

MHU

1. “Mobile health unit” OR “mobile health units” OR “mobile clinic” OR “mobile clinics”
2. “Mobile medical team” OR “Mobile medical teams” OR "mobile health clinic" OR "mobile health clinics" OR "mobile health team" OR "mobile health teams"
3. “Foreign medical team” OR “foreign medical teams” OR “emergency medical team” OR “emergency medical teams” OR “EMT type 1”

Armed conflicts

1. “Armed conflict” OR “armed conflicts” OR “war” OR “wars” OR “war zone” OR “war zones” OR “conflict affected”
2. “conflict zone” OR “conflict zones” OR “conflict setting” OR “conflict settings” OR “complex emergencies”

For the FCAS terms, the MHU terms were combined into two groups and paired with each FCAS term separately.

MHU

1. “Mobile health unit” OR “mobile health units” OR “mobile clinic” OR “mobile clinics” OR “Mobile medical team” OR “Mobile medical teams” OR "mobile health clinic" OR "mobile health clinics"
2. "mobile health team" OR "mobile health teams" OR “Foreign medical team” OR “foreign medical teams” OR “emergency medical team” OR “emergency medical teams” OR “EMT type 1”

For MHU and armed conflict pairings, the first 300 hits of each search were screened. For pairing of MHU and FCAS terms, the first 100 hits of each search were screened due to the high overlap and fewer relevant results per search. All results were saved and exported to EndNote for reference management.
